# Glycosyltransferase GLT8D2 Positively Regulates ApoB100 Protein Expression in Hepatocytes

**DOI:** 10.3390/ijms141121435

**Published:** 2013-10-29

**Authors:** Hong-Shan Wei, Hong-Lian Wei, Fei Zhao, Le-Ping Zhong, Yu-Tao Zhan

**Affiliations:** 1 Institutes of Infectious Disease, Beijing Ditan Hospital, Capital Medical University, Beijing 100015, China; E-Mail: drwei@ccmu.edu.cn; 2 Seventh Department of Internal Medicine, Linyi People’s Hospital, Linyi 276000, Shandong, China; E-Mail: elsevierwhl@163.com; 3 Department of Gastroenterology, Beijing Tongren Hospital, Capital Medical University, Beijing 100730, China; E-Mails: fayefly.future@163.com (F.Z.); zhongleping@gmail.com (L.-P.Z.)

**Keywords:** non-alcoholic fatty liver disease, glycosyltransferase, GLT8D2, apoB100

## Abstract

Non-alcoholic fatty liver disease (NAFLD) is characterized by triglyceride (TG) accumulation in hepatocytes. Very low density lipoprotein (VLDL) is a major secretory product of the liver that transports endogenously synthesized TG. Disrupted VLDL secretion may contribute to the accumulation of TG in hepatocytes. ApoB100 (apolipoprotein B100) is a glycoprotein and an essential protein component of VLDL. Its glycosylation may affect VLDL assembly and secretion. However, which glycosyltransferase catalyzes apoB100 glycosylation is unknown. In this study, we cloned the *GLT8D2 (*glycosyltransferase 8 domain containing 2) gene from HepG2 cells and generated a series of plasmids for *in vitro* studies of its molecular functions. We discovered that GLT8D2 was localized in the ER, interacted with apoB100, and positively regulated the levels of apoB100 protein in HepG2 cells. Based on these results, we propose that GLT8D2 is a glycosyltransferase of apoB100 that regulates apoB100 levels in hepatocytes.

## 1. Introduction

Non-alcoholic fatty liver disease (NAFLD) has become the most common chronic liver disease in the United States and other western countries [1,2]. It is characterized by triglyceride (TG) accumulation in hepatocytes [3,4]. However, the mechanism of this triglyceride accumulation remains elusive. Very low density lipoprotein (VLDL) is a major secretory product of the liver that transports endogenously synthesized lipids, mainly TG [5]. It was recently reported that the secretion rate of VLDL increased linearly with increasing intra-hepatic TG disposal, while that in NAFLD patients did not [6]. Therefore, disrupted VLDL secretion may contribute to the accumulation of triglyceride in hepatocytes. VLDL consists of cholesterol, phospholipids, triglycerides and apolipoproteins that include apoB100, apoC, and apoE [7]. Based on the complexity of VLDL, We speculated that abnormalities in VLDL assembly may be the main cause of VLDL secretion disruption in NAFLD patients.

ApoB100, the essential protein component of VLDL [8], is a glycoprotein. Glycosylation aids the folding of nascent polypeptide chains and stabilizes mature glycoprotein’s conformation [8]. Therefore, glycosylation may affect apoB100’s folding and maturation, which further affects VLDL assembly and secretion. Glycosylation is catalyzed by glycosyltransferase enzymes, and over 160 human glycosyltransferase genes have been cloned [9,10]. However, which glycosyltransferase catalyzes apoB100 glycosylation is unknown. Recently, we cloned a new glycosyltransferase gene, namely GLT8D2. In the present study, we observed that GLT8D2 interacted with apoB100, and this interaction affected apoB100 protein expression in hepatoma cell line (HepG2 cells). These results suggested that GLT8D2 may be a glycosyltransferase of apoB100.

## 2. Results

### 2.1. Amplification of *GLT8D2* Gene

**Figure 1:**
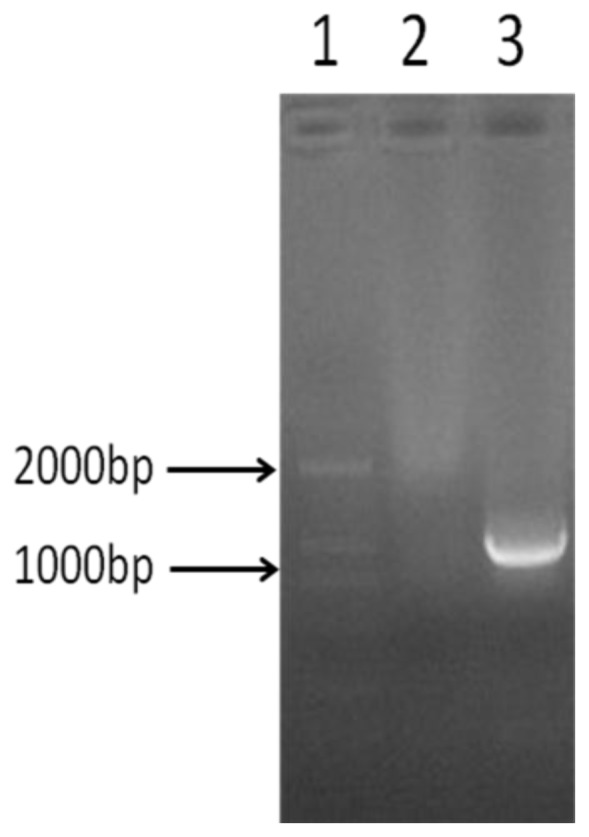
Electrophoresis of GLT8D2 PCR product on 1% agarose gel. **Lane 1**: DNA marker; **Lane 2**: Negative control; **Lane 3**: amplified *GLT8D2* gene (1050 bp).

The *GLT8D2* gene is amplified by using the GLT8D2 cDNA as template and two specific primers. As shown in Figure 1, the GLT8D2 PCR product was approximately 1050 bp, as expected.

### 2.2. Successful Construction of Plasmids

**Figure 2:**
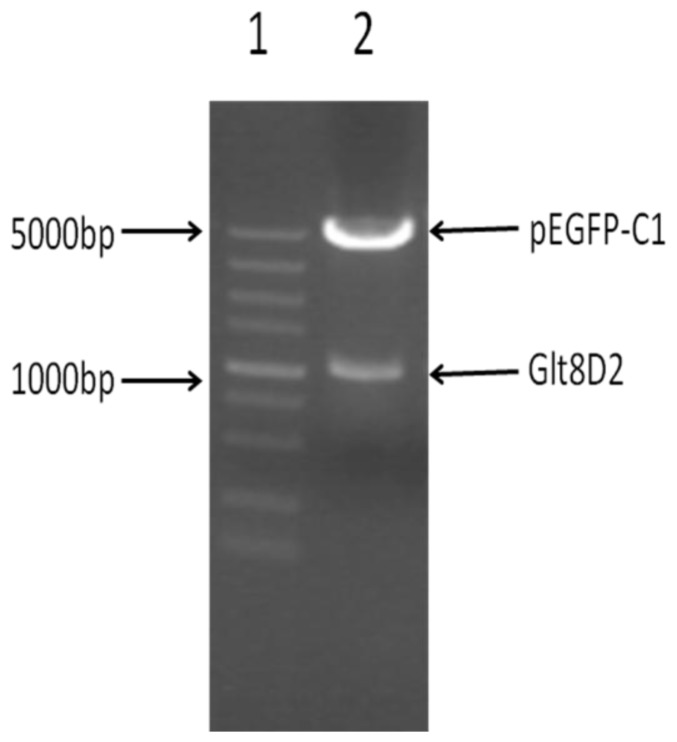
Electrophoresis of recombinant GLT8D2 on 1% agarose gel. **Lane 1**: DNA marker; **Lane 2**: Double digestion of recombinant pEGFP-C1-GLT8D2 with *Kpn*I and *Xho*I restriction enzymes (pEGFP-C1, 4700 bp and GLT8D2, 1050 bp).

The amplified and purified *GLT8D2* gene fragment was ligated into the pEGFP-C1 vector to construct the pEGFP-C1-GLT8D2 plasmid. The integrity of recombinant vector pEGFP-C1-GLT8D2 was confirmed by double restriction enzyme digestion with *Kpn*I and *Xho*I. As shown in Figure 2, two expected bands, one at 4700 bp and another at 1050 bp, were observed after the digestion. Similarly, we constructed the plasmid of pEGFP-C1-mut-GLT8D2 by ligating the purified mutant *GLT8D2* gene fragment into the pEGFP-C1 vector, which was also confirmed by restriction enzyme digestion and electrophoresis (data not shown). The success of plasmid construction was further confirmed by sequencing the inserted gene fragments in pEGFP-C1-GLT8D2 and pEGFP-C1-muta-GLT8D2.

### 2.3. Knockdown Efficiency of pcDNA6.2-GLT8D2 shRNAs in HepG2 Cells

**Figure 3:**
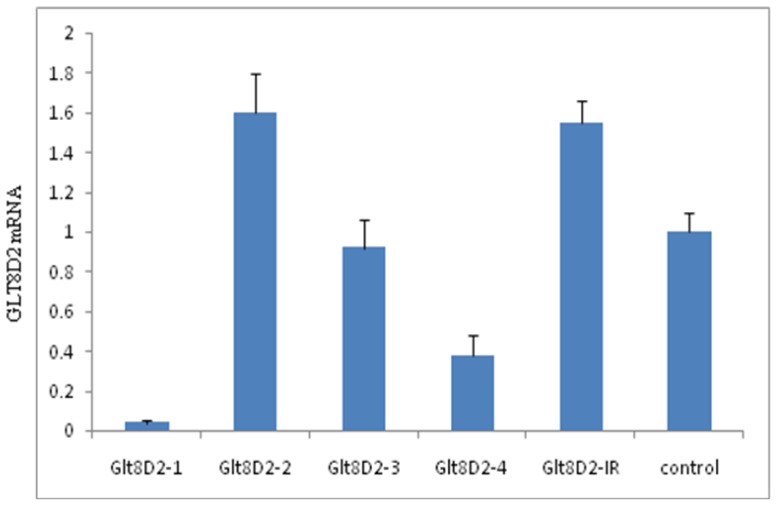
Knockdown efficiency of GLT8D2 shRNAs in HepG2 cells revealed by qRT-PCR.

The pcDNA6.2-GLT8D2 shRNA-1–4, pcDNA6.2-GLT8D2 shRNA-IR, and pcDNA6.2-negative were transfected into HepG2 cells. As shown in Figure 3, only pcDNA6.2-GLT8D2 shRNA-1 and -4 led to significant reduction in the mRNA expression of GLT8D2 (*p* < 0.001 and 0.01 *versus* control, respectively), with pcDNA6.2-GLT8D2 shRNA-1 showing about 94% knockdown efficiency. pcDNA6.2-GLT8D2 shRNA-3 did not affect the mRNA expression of GLT8D2 (*p* > 0.05 *versus* control). pcDNA6.2-GLT8D2 shRNA-2 and pcDNA6.2-GLT8D2 shRNA-IR led to significant increases in the mRNA expression of GLT8D2 (both *p* < 0.05).

### 2.4. Intracellular Localization of the GLT8D2 Protein

**Figure 4:**
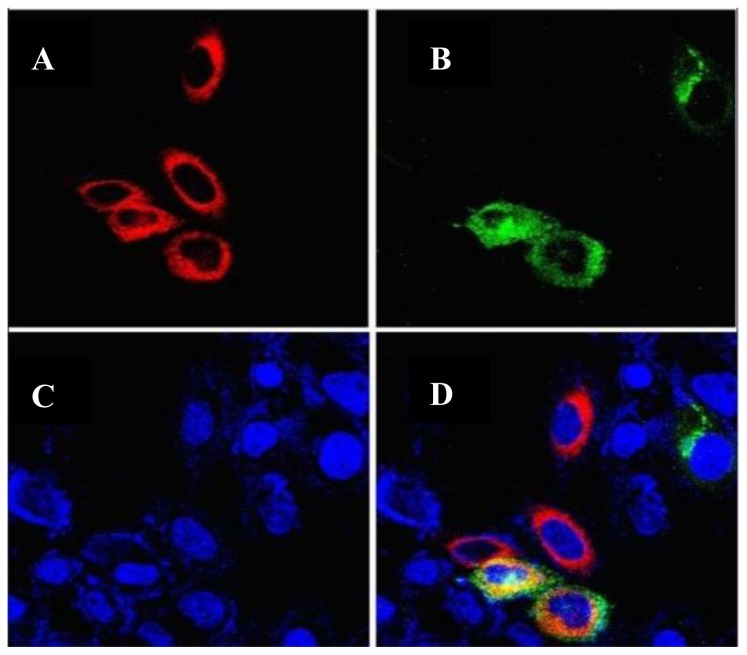
Co-localization of GLT8D2 and calreticulin in HepG2 cells. (**A**) Calreticulin with red fluorescence; (**B**) GLT8D2 with green fluorescence; (**C**) Nuclear with blue fluorescence; (**D**) Calreticulin and GLT8D2 merge with yellow fluorescence.

The plasmids of pEGFP-C1-GLT8D2 and pDsRED-N1-calreticulin were co-transfected into HepG2 cells, and the expressed GLT8D2 and calreticulin were detected by confocal microscopy. The EGFP-C1-GLT8D2 fusion protein emits green fluorescence, the DsRED-N1-calreticulin fusion protein emits red fluorescence, and the DAPI emits blue fluorescence. Double- fluorescence analysis showed that the expression of GLT8D2 (green) in HepG2 cells overlapped with calreticulin (red) (Figure 4), indicating that GLT8D2 protein is localized in the ER around the nucleus in HepG2 cells.

### 2.5. Direct Interaction of GLT8D2 and Apo-B100

**Figure 5:**
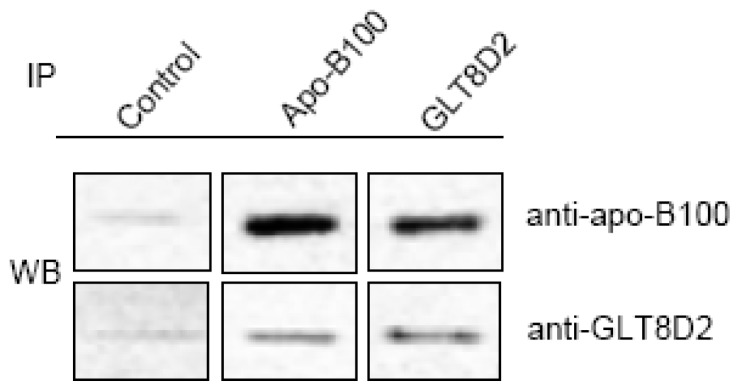
GLT8D2 may be interacted with apo-B100 in HepG2 cells. The result showed co-immunoprecipitation of apo-B100 and GLT8D2 in extracts of HepG2 cells. Anti-GLT8D2 immunoprecipitation (IP) followed by anti-apo-B100 Western blotting (WB) or anti-apo-B100 IP followed by anti-GLT8D2 Western blot. The rabbit irrelevant IgG was used as immunoprecipitated control.

To determine whether GLT8D2 binds to apoB100, co-IP was carried out with HepG2 cell lysate and specific antibodies against GLT8D2 and apoB100. As shown in Figure 5, the GLT8D2 protein was co-immunoprecipitated with the apoB100 protein by the anti-apoB100 antibody, suggesting that GLT8D2 and apoB100 form a complex in HepG2 cells. We observed the molecular weight of GLT8D2 that was consistent with its expected size of 55 kDa.

### 2.6. GLT8D2 Affects ApoB100 Expression in HepG2 Cells

**Figure 6:**
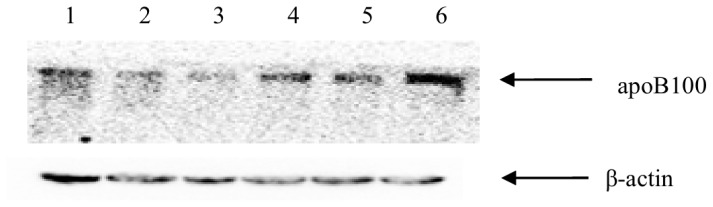
Western blot analysis of apoB100 expression in HepG2 cells with changed expression of GLT8D2. **Lane 1**: pEGFP-C1-GLT8D2; **Lane 2**: pcDNA6.2-GLT8D2-shRNA-1; **Lane 3**: pEGFP-C1-muta GLT8D2; **Lane 4**: pEGFP-C1 empty vector; **Lane 5**: pcDNA6.2-GLT8D2 IR; **Lane 6**: Control.

To determine the effects of GLT8D2 on the expression of apoB100, we over-expressed GLT8D2 by transfecting the GLT8D2 expression plasmid, pEGFP-C1-GLT8D2, and knocked-down the expression of GLT8D2 by transfecting pcDNA6.2-GLT8D2 shRNA-1 in HepG2 cells. We also introduced the mutant GLT8D2 into HepG2 cells by transfecting the GLT8D2 mutant plasmid, pEGFP-C1-mut-GLT8D2. As shown in Figure 6, the expression of apoB100 protein was increased by GLT8D2 overexpression in HepG2 cells; the apoB100 level was also reduced with silenced expression of GLT8D2. Expression of the mutant GLT8D2 in HepG2 cells led to down-regulated expression of apoB100. These results suggested that the expression level of GLT8D2 is positively correlated with that of apoB100 in HepG2 cells.

## 3. Discussion

As a member of the glycosyltransferase 8 family, GLT8D2 is a 349 amino acid single-pass type II membrane protein encoded by a gene that is located on human chromosome 12q23.3. The first six amino acid residues extend to the cytoplasm; the No. 7–24 amino acid residues are in the plasma membrane; the amino acid residues No. 25–349 are in luminal compartments. GLT8D2 is also a glycoprotein with only one glycosylation site, Asn^234^[11]. In this study, we successfully amplified the *GLT8D2* gene and constructed a series of GLT8D2 plasmids, providing valuable materials for future studies on GLT8D2.

The endoplasmic reticulum (ER) plays an essential role in the folding and processing of newly synthesized secretory membrane proteins, which is strictly calcium-dependent [12]. The ER is also crucial for glycoprotein glycosylation [13]. In this study, we observed the localization of GLT8D2 glycosyltransferase in the ER, consistent with its expected role in glycoprotein glycosylation.

ApoB100 is a large secretory glycoprotein with 4536 amino acid residues, and its molecular weight has been calculated to be 513 kDa [14]. However, the glycosyltransferase that catalyzes apoB100 glycosylation is still unknown. It was suggested by Ihara *et al.* that N-Acetylglucosaminyltransferase III may be related to apoB100’s glycosylation in hepatocytes [15]. However, no direct evidence for the interaction between *N*-Acetylglucosaminyltransferase and apoB100 was provided in that study. By using co-immunoprecipitation, we found that GLT8D2 protein was bound to apoB100, suggesting that GLT8D2 may be a glycosyltransferase of apoB100.

ApoB100 is synthesized in hepatocytes. Newly synthesized apoB100 is translocated across the rough endoplasmic reticulum membrane into the lumen, where apoB100 is in at least two pools: a heavy pool, most of which is degraded *in situ*, and a lighter pool, which moves from the rough endoplasmic reticulum lumen through the secretory compartments to the trans-Golgi; it is then packaged with lipid and secreted as VLDL [16]. As a result, degradation and secretion play an important role in the quality control of the apoB100 protein in hepatocytes. It has been shown that 30%–75% of newly synthesized apoB100 is degraded rapidly within 2–3 h [17,18]. The expression of apoB100 is also regulated post-transcriptionally [19]. Glycosylation is one of the most common post-translational modifications in eukaryotic cells with important roles in glycoprotein maturation and function [20–23]. Disrupted glycosylation of apoB100 may lead to its mis-folding then proteasomal degradation [15,24]. Therefore, the glycosylation of apoB100 may be an important mechanism for its degradation. As a secretary protein, apoB100’s glycosylation may also affect its secretion. Vukmirica *et al.* reported that the inhibition of *N*-linked glycosylation of apoB100 with the chemical inhibitor tunicamycin significantly inhibited apoB100 secretion in rat hepatocytes [25]. Furthermore, mutation in the glycosylation sites of apoB100 resulted in decreased secretion efficiency of apoB [17]. It has been previously suggested that the exit of proteins from the ER is a selective process, in which transport signals present in the cytoplasmic tail of cargo membrane proteins must be recognized by coatomer proteins for their incorporation into the COPII vesicles. Two classes of ER export signals have been described for type I membrane proteins, which are diacidic and the dihydrophobic motifs. Claudio *et al.* proposed that the interaction of Sar1 with the [RK](×)[RK] motif at the CT of members of the glycosyl-transferase family, and probably of other type II mem-brane proteins, is an early event in the selection of these proteins as cargo of COPII-transport vesicles on their way to the Golgi complex [26]. ApoB100 exits the ER in the COPII vesicles [27], thus apoB100 secretion may also be regulated by glycosylation. In this study, we found that changed expression levels of GLT8D2 protein led to similar level changes of apoB100 protein in HepG2 cells. We speculated that: (1) GLT8D2 increases apoB100 glycosylation and reduces apoB100 misfolding and proteasomal degradation; (2) GLT8D2 increases apoB100 secretion by enhancing apoB100 glycosylation and transport from ER to trans-Golgi membrane by stimulating transport signals; (3) the effect of GLT8D2 is stronger in reducing apoB100 degradation than that in increasing apoB100 secretion. Therefore, overall, GLT8D2 positively regulates apoB100 protein expression in HepG2 cells. ApoB100 has 19 potential *N*-glycosylation sites, and 16 asparagine residues of ApoB have been reported to be occupied by oligosaccharides, which are high-mannose type, hybrid type, and monoantennary and biantennary complex type [14,28]. Several Ser/Thr sites may be glycosylated by *O*-gycans. The Glt8D2 is a glycosyltransferase gene, but its donor-substrate and receptor substrate are still unknown thus need further study.

## 4. Experimental Section

### 4.1. Materials

TRIzol^®^ Reagent, vector pcDNA6.2, Protein-G/A Beads and Lipofectamine 2000 reagent were all purchased from Life Technologies (Shanghai, China). DNA Recovery Kit was purchased from Beijing Ding Guo Biotechnology (Beijing, China). TurboFect Transfection Reagent was purchased from Fermentas (Beijing, China). DNA polymerase and T4 DNA Ligase were purchased from Promega (Beijing, China). Plasmid miniprep Kit was purchased from Vigorous Biotechnology (Beijing, China). DNA Restriction Enzymes (*Xho*I and *Kpn*I), RT-PCR Kit, dNTPs, and DAN marker were all purchased from TaKaRa (Beijing, China). DAPI was purchased from Sigma (Beijing, China). Anti-apoB100 antibody was purchased from Santa Cruz Biotechnology (Beijing, China). Anti-GLT8D2 antibody was obtained from the Institute of Infectious Disease, Capital Medical University, China (Beijing, China).

### 4.2. Cell Culture

HepG2 cells were purchased from ATCC and reserved in our laboratory in the Institute of Infectious Disease, Capital Medical University. The Cells were cultured in Eagle’s minimum essential medium (EMEM) supplemented with 10% fetal bovine serum (FBS) in a 5% CO_2_-humidified atmosphere at 37 °C.

### 4.3. Total RNA Extraction and cDNA Amplification

Total RNA was extracted from HepG2 cells by using TRIzol reagent according to the manufacturer’s instructions. Two micrograms of extracted RNA was reverse transcribed into cDNA using 4 μL 5× Exscript™ Buffer, 1 μL oligo dT, 1 μL Random 6mers, 1 μL Exscript™ RTase and 9.8 μL DEPC water in a final volume of 20 μL with enzyme buffer for 15 min at 37 °C. GLT8D2 cDNA was amplified by using a 25 μL reaction mixture containing 14 μL water, 5 μL 10 μM primers (2.5 μL of each), 2.5 μL buffer, 0.5 μL 10 mM dNTPs, 0.5 μL DNA polymerase, and 2.5 μL cDNA. The specific primers used were the forward primer, 5′-GGT ACC ATG GCT CTG TTA CGA AAA ATT AAT C-3′ incorporated with a *Kpn*I restriction enzyme site, and the reverse primer, 5′-CTC GAG AGC TAT GGT GAT TGA GTT TAA ATA TC-3′ incorporated with a *Xho*I restriction enzyme site. The GLT8D2 cDNA was amplified by using 40 cycles of denaturation at 94 °C for 40 s, annealing at 65 °C for 40 s, extension at 72 °C for 2 min, followed by 10 min at 72 °C for the final extension. PCR products were recovered from the gel, purified and sequenced.

### 4.4. Plasmid Construction

#### 4.4.1. Plasmid pEGFP-C1-GLT8D2

The purified GLT8D2 cDNA fragment was ligated into the pEGFP-C1 vector by T4 DNA ligase. Recombinant vector pEGFP-C1-GLT8D2 was transformed into competent *E. coli* DH5α cells. The integrity of the recovered plasmid was confirmed by *Kpn*I and *Xho*I restriction enzyme digestion and sequencing.

#### 4.4.2. Plasmid pcDNA6.2-GLT8D2 shRNA

Based on the multivariate biological information law, GLT8D2 was used as the target gene (GenBank accession No. NM.031302), and 4 pairs of short hairpin RNAs (shRNAs) and one irrelevant sequence (GLT8D2 IR) were designed (Table 1). PcDNA6.2-GLT8D2 shRNA plasmid and pcDNA6.2-GLT8D2 IR plasmid were constructed and identified by Life Technologies.

#### 4.4.3. Plasmid pEGFP-C1-muta-GLT8D2

To create the plasmid carrying a mutant GLT8D2 with changed glycosylation, A699 was replaced with T by site-directed mutagenesis with the primer of 5′-GTG ATT GTT GCC ATC ATG ACA GAA TGG WT-3′. The mutant GLT8D2 was amplified, purified, and ligated into pEGFP-C1 vector by T4 DNA ligase. The recombinant vector pEGFP-C1-mut-GLT8D2 was transformed into competent *E. coli* DH5α cells. The integrity of the recovered plasmid was confirmed by *Kpn*I and *Xho*I digestion and sequencing.

#### 4.4.4. Other Plasmids

The plasmids pEGFP-C1 and pDsRED-N1-calreticulin were reserved in Institute of Infectious Disease, Capital Medical University.

### 4.5. Intracellular Localization of GLT8D2

Confocal microscopy imaging was used to detect the sub-cellular localization of GLT8D2 in HepG2 cells. HepG2 cells cultured on plates were co-transfected with pEGFP-C1-GLT8D2 plasmid (green fluorescent) and pDsRED-N1-calreticulin plasmid (red fluorescent), fixed with 4% formaldehyde 48 h post-transfection, stained with DAPI for 10 min, and examined by laser scanning confocal microscopy.

### 4.6. Co-Immunoprecipitation (Co-IP) and Western Blot (WB)

For the co-IP assay, total proteins were extracted from HepG2 cells by lysis buffer, and incubated with anti-GLT8D2 and anti-apoB100 antibodies overnight at 4 °C in the presence of 50 μL Protein-G/A beads. Beads were collected, washed, and resuspended in equal volumes of 5× SDS loading buffer. The immunoprecipitated proteins were separated by SDS-PAGE and transferred onto PVDF membrane. The membrane was blocked with 5% skim milk, incubated at 4 °C overnight with anti-apoB100 antibody, and incubated with HRP-conjugated secondary antibody. The signals were analyzed using the Imaging System.

### 4.7. Detection of ApoB100 Expression in HepG2 Cells

2 × 10^5^ HepG2 cells were seeded onto 6-well plates and grown overnight at 37 °C. The plasmids pEGFP-C1-GLT8D2, pcDNA6.2-GLT8D2 shRNA-1 (result suggest that pcDNA6.2-GLT8D2 shRNA-1 has the best inhibition effect on GLT8D2 mRNA expression in HepG2.), pEGFP-C1-muta-GLT8D2 and pEGFP-C1 empty vector was transfected into HepG2 cells by using Lipofectamine 2000 reagent according to the manufacturer’s instructions. After 48 h, proteins were extracted from HepG2 cells by lysis buffer and analyzed with Western-blot.

### 4.8. Statistics

Data are expressed as mean ± SEM. Statistical significance was assessed by two-way ANOVA. A difference with *p* value less than 0.05 was considered as statistically significant.

## 5. Conclusions

In this study, we cloned *GLT8D2* gene from HepG2 cells and generated a series of plasmids for *in vitro* studies of its molecular functions. We discovered that (1) GLT8D2 is localized in the ER; (2) GLT8D2 has interaction with apoB100; and (3) GLT8D2 positively regulates the levels of apoB100 protein in HepG2 cells. Based on these results, we propose that GLT8D2 is a glycosyltransferase of apoB100 that regulates apoB100 levels in hepatocytes.

## Figures and Tables

**Table 1:** shRNA Sequences.

shRNA ID		Sequences
shRNA GLT8D2 1	Sence:	5′-TGCTGTCATGTTGGCAACAATCACACGTTTTGGCCACTGACTGACGTGTGATTTGCCAACATGA-3′
	Antisence:	5′-CCTGTCATGTTGGCAAATCACACGTCAGTCAGTGGCCAAAACGTGTGATTGTTGCCAACATGAC-3′

shRNA GLT8D2 2	Sence:	5′-TGCTGTTCCCATGAAACACAATCAGCGTTTTGGCCACTGACTGACGCTGATTGTTTCATGGGAA-3′
	Antisence:	5′-CCTGTTCCCATGAAACAATCAGCGTCAGTCAGTGGCCAAAACGCTGATTGTGTTTCATGGGAAC-3′

shRNA GLT8D2 3	Sence:	5′-TGCTGAAATGCTCCGAATATCTGGCAGTTTTGGCCACTGACTGACTGCCAGATTCGGAGCATTT-3′
	Antisence:	5′-CCTGAAATGCTCCGAATCTGGCAGTCAGTCAGTGGCCAAAACTGCCAGATATTCGGAGCATTTC-3′

shRNA GLT8D2 4	Sence:	5′-TGCTGTACAGAATCACACAGAGGGTCGTTTTGGCCACTGACTGACGACCCTCTGTGATTCTGTA-3′
	Antisence:	5′-CCTGTACAGAATCACAGAGGGTCGTCAGTCAGTGGCCAAAACGACCCTCTGTGTGATTCTGTAC-3′

shRNA GLT8D2 IR	Sence:	5′-TGCTGAAATGTACTGCGCGTGGAGACGTTTTGGCCACTGACTGACGTCTCCACGCAGTACATTT-3′
	Antisence:	5′-CCTGAAATGTACTGCGTGGAGACGTCAGTCAGTGGCCAAAACGTCTCCACGCGCAGTACATTTC-3′
